# Simultaneous integrated boost plan comparison of volumetric‐modulated arc therapy and sliding window intensity‐modulated radiotherapy for whole pelvis irradiation of locally advanced prostate cancer

**DOI:** 10.1120/jacmp.v14i4.4094

**Published:** 2013-07-08

**Authors:** Olivier Riou, Pauline Regnault de la Mothe, David Azria, Norbert Aillères, Jean‐Bernard Dubois, Pascal Fenoglietto

**Affiliations:** ^1^ Radiation Oncology Department Montpellier Cancer Institute Montpellier France; ^2^ Radiation Oncology Department Poitiers University Hospital Poitiers France

**Keywords:** prostate cancer, volumetric‐modulated arc therapy, intensity‐modulated radiotherapy, whole pelvis irradiation, simultaneous integrated boost plan, organs‐at‐risk sparing, target volume coverage

## Abstract

Concurrent radiotherapy to the pelvis plus a prostate boost with long‐term androgen deprivation is a standard of care for locally advanced prostate cancer. IMRT has the ability to deliver highly conformal dose to the target while lowering irradiation of critical organs around the prostate. Volumetric‐modulated arc therapy is able to reduce treatment time, but its impact on organ sparing is still controversial when compared to static gantry IMRT. We compared the two techniques in simultaneous integrated boost plans. Ten patients with locally advanced prostate cancer were included. The planning target volume (PTV) 1 was defined as the pelvic lymph nodes, the prostate, and the seminal vesicles plus setup margins. The PTV2 consisted of the prostate with setup margins. The prescribed doses to PTV1 and PTV2 were 54 Gy in 37 fractions and 74 Gy in 37 fractions, respectively. We compared simultaneous integrated boost plans by means of either a seven coplanar static split fields IMRT, or a one‐arc (RA1) and a two‐arc (RA2) RapidArc planning. All three techniques allowed acceptable homogeneity and PTV coverage. Static IMRT enabled a better homogeneity for PTV2 than RapidArc techniques. Sliding window IMRT and VMAT permitted to maintain doses to OAR within acceptable levels with a low risk of side effects for each organ. VMAT plans resulted in a clinically and statistically significant reduction in doses to bladder (mean dose IMRT: 50.1±4.6Gy vs. mean dose RA2: 47.1±3.9Gy,p=0.037), rectum (mean dose IMRT: 44±4.5 vs. mean dose RA2:41.6±5.5Gy,p=0.006), and small bowel ($V_{30}\text{IMRT}:76.47\pm 14.91\;\%$ vs. $V_{30}\;\text{RA}2:47.49\pm 16.91\;\%,p=0.002$). Doses to femoral heads were higher with VMAT but within accepted constraints. Our findings suggest that simultaneous integrated boost plans using VMAT and sliding window IMRT allow good OAR sparing while maintaining PTV coverage within acceptable levels.

PACS number: 87.53.Jw

## I. INTRODUCTION

Prostate cancer is the most common cancer in males. Concurrent prostate radiotherapy and androgen deprivation has become a standard of care for patients with locally advanced tumors.^(1)^ More particularly, in most prospective trials that have established the efficacy of such combination, patients were treated with whole pelvic irradiation followed by a prostate boost and two to three years of hormonal therapy.^2^, ^3^ Although there is a growing amount of literature on dose escalation for prostate radiotherapy when combined with androgen deprivation in high‐risk patients, no published level I data are currently available.^4^, ^5^, ^6^ Nevertheless, some randomized clinical trials, such as the GETUG 18 trial, are ongoing to address this question.^(7)^ In France, most centers currently treat such patients with tridimensional conformal radiotherapy (3D CRT) delivering 50 Gy to the pelvic lymph nodes and a prostate boost to 74 Gy, resulting in acceptable cure rates but improvable morbidity. Because intensity‐modulated radiotherapy (IMRT) has the ability to deliver highly conformal dose to the target while lowering irradiation of critical organs, it is becoming the standard radiotherapy technique. However, treatment delivery time is significantly prolonged with both step‐and‐shoot and dynamic IMRT.^(8)^ In recent years, volumetric‐modulated arc therapy (VMAT) has surged as an efficient IMRT technique, and is now commercially available. RapidArc is one of those VMAT systems that has been evaluated through comparison studies. Recent reports have demonstrated that the RapidArc technique was able to reduce treatment time and doses to the organs at risk as compared to static gantry IMRT for the treatment of prostate alone, prostate plus seminal vesicles, and postoperative prostate bed.^9^, ^10^, ^11^, ^12^ One study evaluating this technique in locally advanced prostate cancer involving seminal vesicles and pelvic lymph nodes showed a worse organ sparing with RapidArc compared to static gantry IMRT, but this study compared both techniques by a two‐phase planning process (primary plan to the pelvis and additional boost plan to the prostate).^(13)^ Few studies have evaluated simultaneous integrated boost radiation delivery for pelvis irradiation. In that context, the purpose of the present dosimetric study was to evaluate, with our own institutional constraints, the efficiency of VMAT and sliding window IMRT in simultaneous integrated boost plans.

## II. MATERIALS AND METHODS

### A. Patient selection

Ten patients with locally advanced prostate cancer requiring treatment with concurrent radiotherapy and androgen deprivation were included in this study. All of them were high‐risk patients according to the D'Amico classification (PSA>20, T2c or higher or Gleason score 8 to 10), and at risk of pelvic lymph node involvement as evaluated by the Partin tables and Roach formula. A complete evaluation including clinical examination, blood test, abdominal CT scan, and bone scan was performed to exclude metastatic disease.

### B. Acquisition and simulation

Patients underwent CT‐based virtual simulation (CT Simulator, General Electric, Cleveland, OH) with 2.5 mm thick slices obtained at 2.5 mm intervals in the supine position. A small flexible rectal tube was inserted to evacuate flatus and then removed. Intravenous contrast was used in all patients to permit better delineation of pelvic lymph nodes and bladder. Patients were positioned with knee and feet support (Sinmed B.V., Reeuwijk, The Netherlands), but no custom immobilization device was used. The isocenter was set in the middle of the prostate using our virtual simulation console (AdvantageSim; General Electric, Cleveland, OH) by the treating physician immediately after the first scan, while the patient waited in the treatment position on the scan table. The isocenter was then tattooed on the patient skin using our scan room mobile lasers (LAP Dorado CT‐4; LAP, Luneburg, Germany).

### C. Contouring and volume definition

Structures were manually contoured on the CT scan. The clinical target volume (CTV) for the lymph nodes included the obturator, internal iliac, external (excluding the artery) iliac and sacral vessels plus a circumferential 7 mm margin in accordance with the RTOG consensus guidelines for contouring.^(14)^ The planning target volume PTV1 was defined as the prostate and seminal vesicles (proximal part in the absence of invasion) plus a 1 cm margin in all directions, except posteriorly (5 mm), plus the CTV for lymph nodes and a 7 mm setup margin. The PTV2 consisted of the prostate gland only or the prostate gland plus invaded seminal vesicles, with a 1 cm (5 mm posteriorly) margin. The bladder was contoured in its entirety. The rectum was contoured as a whole organ but starting 2 cm above and below the CTV. Femoral heads were drawn from the top of the acetabulum to the small trochanter inferiorly. Small bowel was determined in all slices where the PTV was apparent. To take interfractional bowel motion into account, the small bowel was delineated as a whole pelvic and abdominal cavity excluding bones, muscle, and other organs at risk (OAR), rather than contoured as individual bowel loops. For bowel, rectum, and bladder, a second volume was created and defined as the considered organ minus the PTV (bowel ‐ PTV, rectum ‐ PTV, bladder ‐ PTV) to avoid hot spots and improve optimization.

### D. treatment planning and optimization process

#### D.1 Dose prescription

The prescribed doses to the PTV1 and PTV2 were, respectively, 54 Gy and 74 Gy in 37 daily fractions.

#### D.2 IMRT

Treatment plans were generated using commercial software (Eclipse, Helios, version 8.2.23; Varian Medical Systems, Palo Alto, CA). Beam geometry consisted of seven coplanar split fields with gantry angles of 0°, 45°, 110°, 165°, 195°, 250°, and 325°. IMRT was delivered using an 18 MV linear accelerator (21 EX; Varian Medical Systems) and the “sliding window” mode of the multileaf collimator (MLC Millennium 120; Varian).

Calculation was performed with AAA algorithm and grid of 2.5 mm.

#### D.3 RA

RapidArc plans (Varian Medical Systems) were performed using the Eclipse software version 8.9.08 (Helios; Varian Medical Systems). A maximum dose rate of 600 MU/min and 18 MV photon beams were selected. RapidArc with one arc (RA1) corresponded to a single 360° rotation and RapidArc with two arcs (RA2) to two coplanar arcs of 360° sharing the same isocenter and optimized independently and simultaneously. These two arcs were delivered with opposite rotation (clockwise and counterclockwise), so that off‐treatment between the two beams was minimized to about 25 seconds. For RA1, field size and collimator rotation were determined by the automatic tool from Eclipse to encompass the PTV. We ensured that the collimator was rotated to a value different from zero in order to avoid the tongue‐and‐groove effect. For RA2, the first arc was similar to that defined in the RA1 process, except for the rotation of the collimator which was 360‐X for the second arc (X corresponded to the rotation of the collimator of the first arc).

#### D.4 Optimization process and constraints

Optimization process was undertaken by decreasing as much as possible the dose to OAR without altering PTV coverage, and the results were improved by modifying constraints and priority factors. These parameters were modified with regard to the DVH results for each patient. We always checked that a good PTV2 coverage by the 74 Gy isodose was obtained, 95% of the PTV receiving at least 95% of the prescribed dose. The same dosimetric constraints for OAR were used for IMRT and VMAT. Regarding rectal dose, the maximal dose allowed was 74 Gy; the volume receiving 72 Gy ($V_{72}$) should remain less than 25% and the $V_{60}$ less than 50%. For bladder, the maximal dose was 74 Gy; $V_{70}$ should remain less than 50% and $V_{75}$ less than 25%. The maximal dose to the femoral heads was 55 Gy and $V_{50}$ was less than 5%. The maximal dose allowed for small bowel was 34 Gy, with less than 450 cc receiving 30 Gy or more and less than 200 cc receiving 40 Gy or more.

IMRT and VMAT use different optimization algorithms and processes. IMRT plans were always tuned to achieve the best result for a specific patient and the best plan that we could obtain was the one presented in the study. All the parameters provided in the IMRT optimizer software were used, always keeping the same geometry and number of fields. VMAT plans with one arc or two arcs were optimized to try to achieve the best results with the possibility offered by the software. In fact, the comparison was done between “the best plans obtained with the specific technique”. For each individual patient, the same normalization was used for the RA and IMRT plans.

### E. Statistical analysis

Doses to the PTV1, PTV2, and OAR were recorded for sliding window IMRT and RapidArc plans. A nonparametric Wilcoxon matched pair test was used for comparison between values of IMRT and RapidArc for organs at risks, PTV1, and PTV2. A two‐tailed p‐value less than 0.05 was used to indicate statistical significance.

## III. RESULTS

### A. Contoured volumes

The mean contoured volumes (in cc±standard deviation(SD), minimum and maximum values in brackets) were: PTV1:906±355(419–1356); PTV2:151±56(94–290); small bowel: 342±145(163–581); bladder: 129±64(37–239); rectum: 58±19(31–71).

### B. PtV coverage

The doses received by 95% of the $\text{PTV}1(\text{PTV}1\;D_{95})$ were 53.3±0.9Gy,53.7±0.9Gy and 53.7±1.3Gy, respectively, for IMRT, RA1, and RA2. This means that all three techniques allowed a good PTV1 coverage by the 95% isodose of the prescribed dose (51.3 Gy). Mean doses to PTV1 were 60.3±2.1Gy,61.6±2Gy, and 61.8±2Gy, respectively, for IMRT, RA1, and RA2.

Mean doses to PTV2 were 73.1±0.8Gy,74.1±0.9Gy, and 74.5±0.6Gy, respectively, for IMRT, RA1, and RA2. Maximal doses received by the PTV2 were 75.5±1.2Gy,77.8±1.1Gy, and 77.4±0.8Gy, respectively, for IMRT, RA1, and RA2, which is less than 106% of the prescribed dose (78.4 Gy).

**Figure 1:**
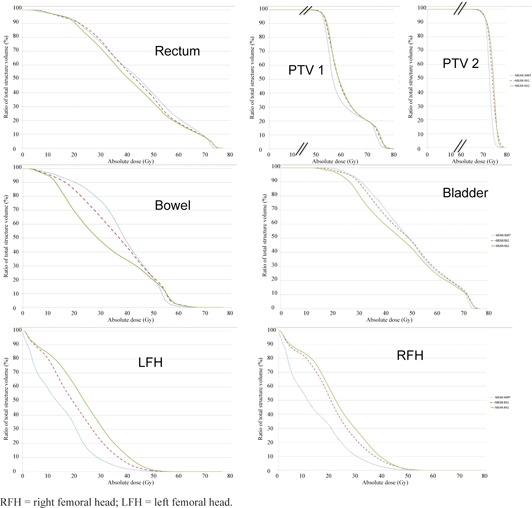
Mean OAR and PTV DVH plots for the ten patients with IMRT (in dashed blue), RA1 (in dashed red), RA2 (in green), x‐axis in gray, and y‐axis in percentage of the corresponding volume.

Figure 1 shows dose‐volume histograms obtained with the three techniques for PTV1 and PTV2.

**Figure 2:**
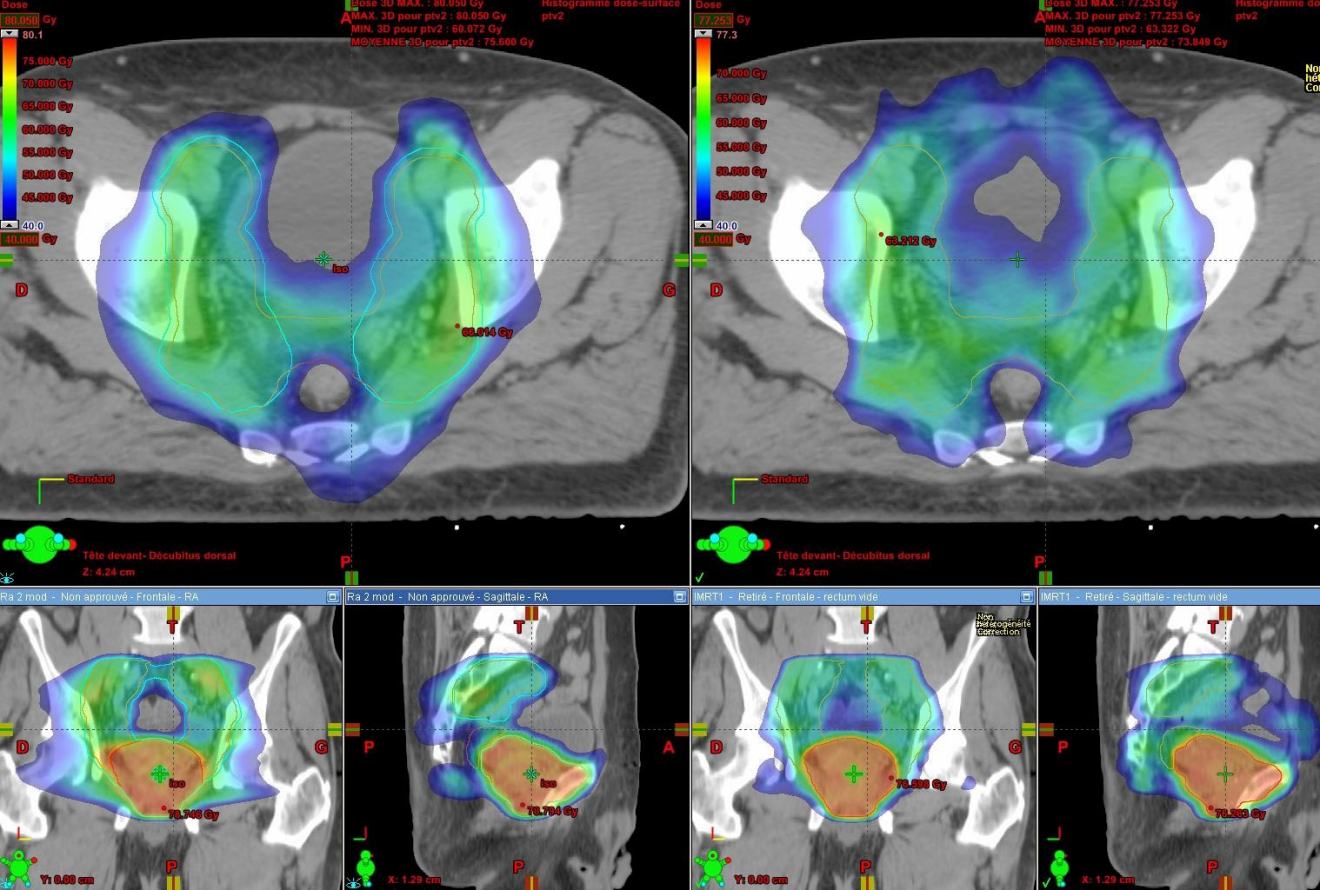
Typical dosimetric results for static IMRT (on right) and RapidArc (on left) plans in the axial, coronal, and sagittal views. Colowash isodose lines are from 40 Gy to maximal dose.

Figure 2 represents typical dosimetric results for static IMRT and RapidArc plans.

### C. Organs‐at‐risk sparing

Figure 1 shows dose‐volume histograms obtained with the three techniques for bladder, small bowel, rectum, and femoral heads.

All three techniques permitted to maintain doses to OAR within dose levels recommended by the QUANTEC, with a low risk of side effects.^(15)^

For bladder, although VMAT induced a slight increase of the maximal dose corresponding to hot points inside the PTV overlapping the bladder, there was a substantial gain in the middle doses, with mean bladder $V_{45}$ values (defined as the mean percentage volume receiving 45 Gy or more, SD in brackets) of 60.0% (±12%) for IMRT, 57.5% (±11%) for RA1, and 52.4% (±8%) for RA2.

Femoral heads were more irradiated with both one‐ and two‐arc RapidArc plans than with the IMRT plan. Mean dose values (SD in brackets): (i) for the right femoral head were 14.4(±2.4)Gy for IMRT, 20.9(±4.0)Gy for RA1, and 22.8(±5.4)Gy for RA2; (ii) for the left femoral head 14.5(±1.6)Gy for IMRT, 20.3(±2.9)Gy for RA1, and 23.5(±5.2)Gy for RA2.

Both VMAT plans produced higher maximal rectal dose (hot points inside the PTV overlapping the rectum), but for all the dose levels below 71 Gy, this difference was in favor of VMAT with on average a 4 Gy benefit for the median dose with RA2 as compared to static IMRT.

A significant advantage for RapidArc was seen for the small bowel with a 2.3 Gy and a 6.4 Gy benefit on mean dose, respectively, for RA1 and RA2 as compared to IMRT. On average, the volume of small bowel receiving 30 Gy or more ($V_{30}$) was 48 cc and 100 cc smaller, respectively, with RA1 and RA2 than with IMRT.

Table 1 summarizes the main and most significant results obtained with the three techniques.

**Table 1:** Dosimetric results for bladder, rectum, small bowel, and monitor units with the three techniques

*OAR/MU*	*Bladder*			*Rectum*				*Small Bowel*					*MU*
*Dosimetric Parameter*	*Mean Dose (Gy)*	$V_{60}\;(\%)$	$V_{70}\;(\%)$	*Mean Dose (Gy)*	$V_{50}\;(\%)$	$V_{60}\;(\%)$	$V_{70}\;(\%)$	*MeanDose (Gy)*	$V_{30}\;(\%)$	$V_{30}\;(\text{cc})$	*V40 (%)*	*V40 (cc)*	
IMRT	50.1±4.6	27.1±12.1	14±7.3	44±4.5	44.5±5.6	21.6±9.2	8.8±6.6	38.0±5.3	76.5±14.9	262±22	46.6±16.3	160±24	1297±166
RA1	49.3±4.7	28.3±12.0	14.6±8.0	42.9±4.7	43.6±5.2	18.4±9.5	8.2±7.2	35.7±5.9	62.4±14.7	214±21	41.5±13.4	142±19	512±66
P‐value IMRT vs. RA1	0.492	0.0645	0.1055	0.0488^a^	0.2754	0.0039^a^	0.2969	0.0371^a^	0.0059^a^		0.0645		0.002^a^
RA2	47.1±3.9	25.2±9.6	14.1±8.7	41.6±5.5	40.8±6.6	17.7±9.1	8.1±6.4	31.6±6.9	47.5±16.9	162±24	33.9±13.8	116±20	452±71
P‐value IMRT vs RA2	0.037^a^	0.1934	0.9999	0.006^a^	0.0059^a^	0.0020^a^	0.0645	0.002^a^	0.002^a^		0.0039^a^		0.002^a^

a A statistically significant difference in favor of the RapidArc treatment.

### D. Efficiency

RapidArc techniques induced almost a threefold decrease in the number of monitor units (MU) delivered. Of note, the number of MUs is not increased when adding a second arc. Values and statistical analysis for MUs are shown in Table 1.

### E. Integral dose and whole body irradiation

**Figure 3:**
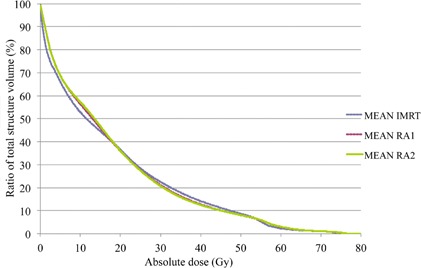
Dose‐volume histogram for the whole body (integral dose) for the ten patients with IMRT (in dashed blue), RA1 (in dashed red), RA2 (in green), x‐axis in gray (Gy), and y‐axis in percentage of the corresponding volume.

Figure 3 represents a dose‐volume histogram for the whole body. VMAT delivers slightly more low doses (0 to 18 Gy) while sliding window IMRT irradiates more volume at higher doses (18 to 54 Gy), but the histograms are quite similar in shape and differences are mild.

## IV. DISCUSSION

Dose levels and treatment volumes remain controversial topics for high‐risk prostate cancer radiotherapy.^16^, ^17^ However, whole pelvic irradiation is often considered in this setting, raising concerns about an increase in radiation‐related toxicity. New technical developments have allowed radiation oncologists to achieve a better protection of critical organs while providing higher dose conformity to target volumes. More specifically, several studies indicated that VMAT treatment offers equal or better dosimetric results compared to static gantry IMRT when treating target volumes including the prostate gland alone or the prostate gland plus seminal vesicles.^9^, ^10^, ^11^, ^12^ On the contrary, only two studies have directly compared VMAT and IMRT treatment plans with pelvic lymph nodes irradiation in high‐risk prostate cancer patients.

Davidson et al.^(18)^ have recently published a study assessing the role of VMAT relative to IMRT and helical tomotherapy (HT) in the management of different clinical scenarios: localized, locally advanced or postoperative prostate cancer. They found that VMAT was able to improve efficiency of delivery while maintaining equivalent dosimetric quality as compared to IMRT and HT. However, this study by its nature, dealt with various clinical conditions and yields potentially confusing results. Indeed, for each condition, the subgroups were composed of only five patients, making definitive conclusions difficult.^(19)^

Yoo et al.^(13)^ reported the results of a dosimetric study comparing the treatment plans of ten patients with PTV including prostate, seminal vesicles, and lymph nodes. With a two‐phase planning process (primary plan to the pelvis and additional boost plan to the prostate), Yoo and colleagues showed that IMRT reached better dose sparing for bladder, rectum, and small bowel than did RapidArc.

By contrast, our results indicate a clinically and statistically significant reduction in doses delivered to the bladder, rectum, and small bowel when using RapidArc in simultaneous integrated boost plans. The dose to femoral heads was higher with VMAT, but it remained within accepted constraints and far from any complication dose level. Anyway, we do not believe that the increase in femoral dose explains the decrease in bladder, bowel, and rectum doses for VMAT. For IMRT, the system had the possibility to decrease the dose to bladder and rectum by increasing the femoral dose to the optimization constraint that we set, but it didn't because it resulted in no gain for bladder, bowel, and rectal protection. The geometric arrangement and number of beams have an impact on the dosimetric results. Probably we could obtain better results with IMRT by increasing the number of beams, but using more than seven split fields would result in a dramatic increase in delivery time, not compatible with pelvic irradiation.

A common explanation for different dosimetric results in the same setting is the variation in the definition of volumes. Nevertheless, we do not believe this could justify the major difference between these results, because our patients had overall larger PTV and smaller OAR volumes than had the patients treated in the aforementioned study which led to even more complicated dosimetric plans. For example, the small bowel was delineated only on the slices where the PTV was present in our patients, whereas in the Yoo et al. study it was contoured up to 3 cm above the most superior slice of the PTV, where obviously there is almost no radiation dose. Another explanation could be that we focused on improving OAR sparing, even if it led to a slight increase in PTV heterogeneity, provided this heterogeneity remained between acceptable levels for each patient as defined by the ICRU 83 recommendations.^(20)^

The last explanation could be that we used a simultaneous integrated boost method to deliver the dose to the prostate and pelvis, while Yoo and colleagues used a primary plan and a separate boost plan for each patient in their study. A single‐phase process has been shown to give better results than a two‐phase plan to simultaneously deliver high dose to the prostate and lower dose to the pelvic nodes in high‐risk prostate cancer when using IMRT.^(21)^ This has not been done for VMAT and the only way to answer the question will be to undertake another dosimetric study comparing simultaneous integrated boost with two‐phase plans with this technique. One could criticize this single‐phase approach, pointing out that the delivery of different dose levels with the same number of fractions necessarily leads to a change in the fractionation regarding the different target volumes. The way to overcome this limitation is either to hypofractionate the prostate volume while keeping a 1.8 or 2 Gy/fraction regimen on the pelvic volume, or to maintain a standard fractionation for the prostate volume while reducing the dose per fraction to the pelvis. We chose the second solution in order to achieve a lower rate of toxicity. We must assume a high degree of uncertainty regarding the α/β ratio for prostate cancer and the sensitivity of prostate tumors to fractionation, making biologically equivalent dose calculations hazardous.^22^, ^23^, ^24^ The PTV1 dose prescription was 54 Gy in 37 fractions corresponding to 1.45 Gy per fraction. Considering an α/β ratio of 3, the equivalent total dose for a 2 Gy fractionation would be 48 Gy and with an α/β ratio of 4, the equivalent total dose for a 2 Gy fractionation would be 49 Gy. This fractionation has been used in clinical practice in our institution for pelvic IMRT since 2000, and we have not observed a worsening tumor control so far (unpublished data).

Not surprisingly, two‐arc plans provided better results both in terms of organ sparing and PTV coverage than single‐arc plans. However, both plans were acceptable. Clearly, the overall treatment delivery time is longer when adding a second arc, but far inferior to static IMRT delivery, emphasizing the need for clinicians to take this factor into consideration when choosing the most appropriate treatment plan for each individual patient.

## V. CONCLUSIONS

This study, performed on ten patients, indicates that simultaneous integrated boost plans using VMAT and sliding window IMRT allows good OAR sparing while maintaining PTV coverage within acceptable levels for whole pelvis irradiation of locally advanced prostate cancer. RapidArc improves treatment efficiency thanks to a dramatic fall in the number of MU used for irradiation.

## ACKNOWLEDGMENTS

We thank Vanessa Guillaumon (Research department, Montpellier Cancer Institute, Montpellier, France) who provided medical writing services.

## Supporting information

Supplementary Material

Supplementary Material

Supplementary Material

Supplementary Material
