# Supplementary figures and images for: Simultaneous integrated boost plan comparison of volumetric‐modulated arc therapy and sliding window intensity‐modulated radiotherapy for whole pelvis irradiation of locally advanced prostate cancer
Source: J Appl Clin Med Phys. 2013 Jul 8;14(4):26–35. doi: 10.1120/jacmp.v14i4.4094 (PMC5714536; doi:10.1120/jacmp.v14i4.4094)

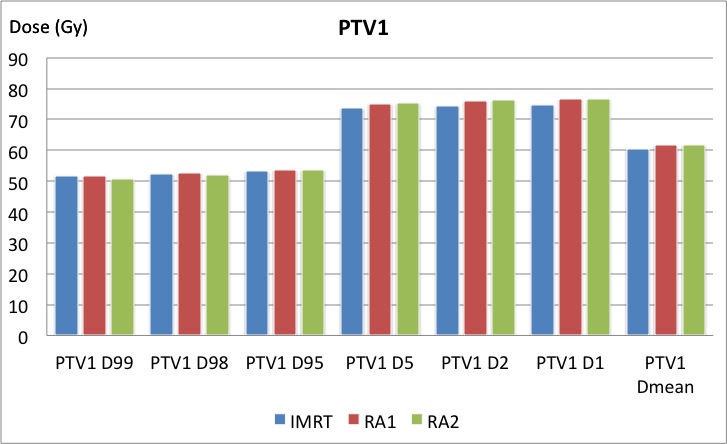

Supplement: Supplementary file 1 — Supplementary Material [file ACM2-14-026-s001.jpg]

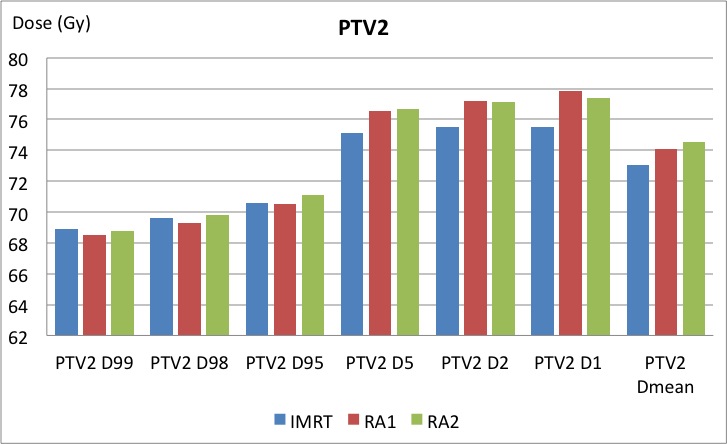

Supplement: Supplementary file 2 — Supplementary Material [file ACM2-14-026-s002.jpg]

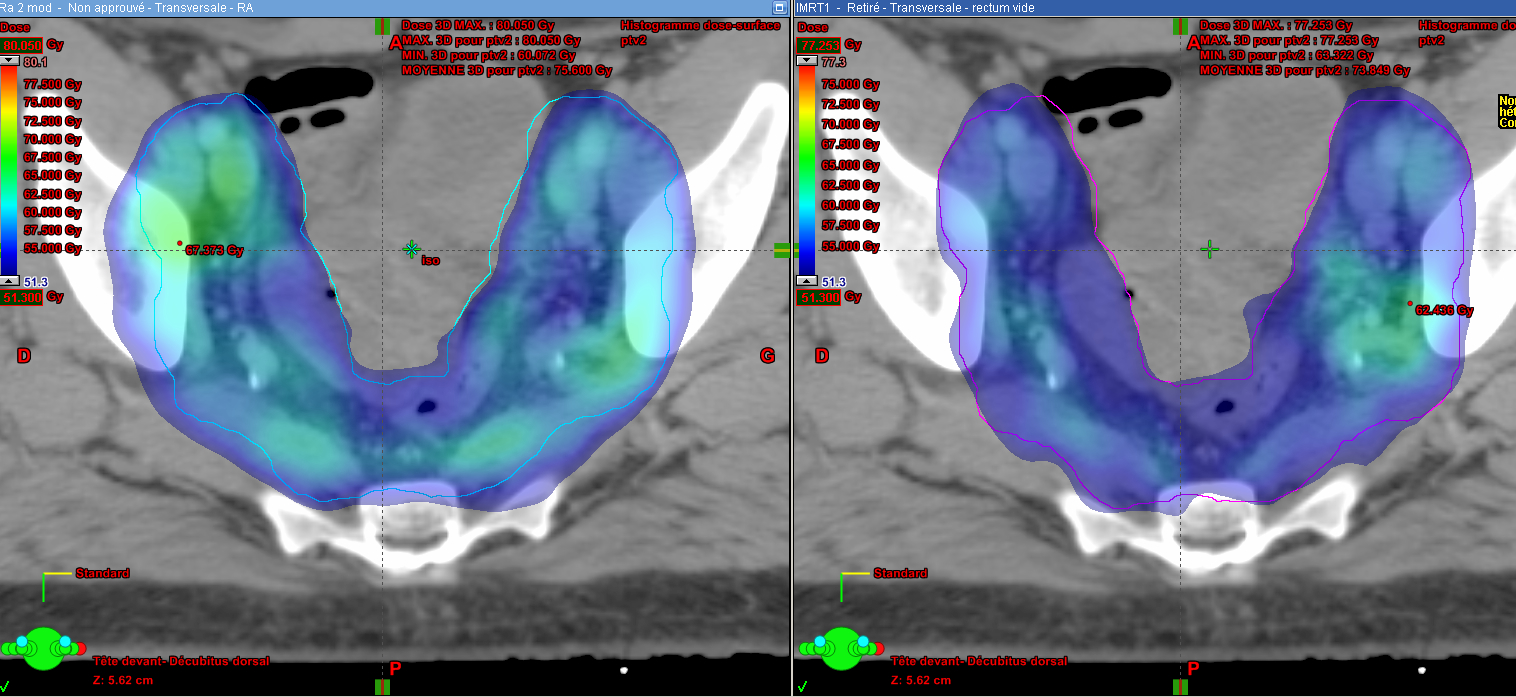

Supplement: Supplementary file 3 — Supplementary Material [file ACM2-14-026-s003.jpg]
