# Supplementary material for: Simultaneous integrated boost plan comparison of volumetric‐modulated arc therapy and sliding window intensity‐modulated radiotherapy for whole pelvis irradiation of locally advanced prostate cancer
Source: J Appl Clin Med Phys. 2013 Jul 8;14(4):26–35. doi: 10.1120/jacmp.v14i4.4094 (PMC5714536; doi:10.1120/jacmp.v14i4.4094)
Supplement: Supplementary file 4 — Supplementary Material [file ACM2-14-026-s004.doc]

Simultaneous integrated boost plan comparison of volumetric modulated arc therapy and sliding-window intensity modulated radiotherapy for whole pelvis irradiation of locally advanced prostate cancer

**Abstract:**

Background: Concurrent radiotherapy to the pelvis plus a prostate boost with long-term androgen deprivation is a standard of care for locally advanced prostate cancer. IMRT has the ability to deliver highly conformal dose to the target while lowering irradiation of critical organs around the prostate. Volumetric modulated arc therapy is able to reduce treatment time but its impact on organ sparing is still controversial when compared to static gantry IMRT.

Methods: 10 patients with locally advanced prostate cancer were included. The planning target volume (PTV) 1 was defined as the pelvic lymph nodes, the prostate, and the seminal vesicles plus set up margins. The PTV 2 consisted of the prostate with set up margins. The prescribed doses to PTV1 and PTV2 were 54 Gy in 37 fractions and 74 Gy in 37 fractions, respectively. We compared simultaneous integrated boost plans by means of either a 7 coplanar static split fields IMRT, or a one-arc (RA1) and a two-arc (RA2) RapidArc® planning.

Results: All three techniques allowed acceptable homogeneity and PTV coverage. Static IMRT enabled a better homogeneity for PTV2 than RapidArc® techniques. Sliding-window IMRT and VMAT permitted to maintain doses to OAR within acceptable levels with a low risk of side-effects for each organ. Nevertheless, VMAT plans resulted in a clinically and statistically significant reduction in doses to bladder (Mean dose IMRT: 50.1 ± 4.6 Gy *vs* Mean dose RA2: 47.1 ± 3.9 Gy, p = 0.037), rectum (Mean dose IMRT: 44 ± 4.5 *vs* Mean dose RA2: 41.6 ± 5.5 Gy, p = 0.006) and small bowel (V30 IMRT: 76.47 ± 14.91% *vs* V30 RA2: 47.49 ± 16.91%, p = 0.002).

Conclusions: Our findings suggest that VMAT allows better OAR sparing as compared to sliding-window IMRT while maintaining PTV coverage within acceptable levels.

**Introduction:**

Prostate cancer is the most common cancer in males. Concurrent prostate radiotherapy and androgen deprivation has become a standard of care for patients with locally advanced tumors [1]. More particularly, in most prospective trials that have established the efficacy of such combination, patients were treated with whole pelvic irradiation followed by a prostate boost and 2-3 years of hormonal therapy [2, 3]. Although there is a growing amount of literature on dose escalation for prostate radiotherapy when combined with androgen deprivation in high-risk patients, no published level I data are currently available [4-6]. Nevertheless, some randomized clinical trials, such as the Getug 18 trial, are ongoing to address this question [7]. In France, most centers currently treat such patients with tridimensional conformal radiotherapy (3D-CRT) delivering 50 Gy to the pelvic lymph nodes and a prostate boost to 74 Gy, resulting in acceptable cure rates but improvable morbidity. Because Intensity modulated radiotherapy (IMRT) has the ability to deliver highly conformal dose to the target while lowering irradiation of critical organs, it is becoming the standard radiotherapy technique. However, treatment delivery time is significantly prolonged with both step-and-shoot and dynamic IMRT [8]. In the recent years, Volumetric Modulated Arc Therapy (VMAT) has surged as an efficient IMRT technique, and is nowadays commercially available. RapidArc® (Varian Medical Systems, Palo Alto, CA) is one of those VMAT systems that has been evaluated through comparison studies. Recent reports have demonstrated that the RapidArc® technique was able to reduce treatment time and doses to the organs at risk as compared to static gantry IMRT for the treatment of prostate alone, prostate plus seminal vesicles, and post-operative prostate bed [9-12]. One study evaluating this technique in locally advanced prostate cancer involving seminal vesicles and pelvic lymph nodes showed a worse organ sparing with RapidArc® compared to static gantry IMRT, but this study compared both technique by a two-phases planning process (primary plan to the pelvis and additional boost plan to the prostate) [13]. In that context, the purpose of the present dosimetric study was to evaluate, with our own institutional constraints, the efficiency of these new techniques in simultaneous integrated boost plans.

**Methods:**

# Patient selection

Ten patients with locally advanced prostate cancer requiring treatment with concurrent radiotherapy and androgen deprivation were included in this study. All of them were high-risk patients according to the D'Amico classification (PSA > 20, T2c or higher or Gleason score 8 to 10), and at risk of pelvic lymph node involvement as evaluated by the Partin tables and Roach formula. A complete evaluation including clinical examination, blood test, abdominal CT scan and bone scan was performed to exclude metastatic disease.

# Acquisition and simulation

Patients underwent CT-based virtual simulation (CT Simulator, General Electric, Cleveland, OH) with 2.5 mm thick slices obtained at 2.5 mm intervals in the supine position. A small flexible rectal tube was inserted to evacuate flatus, and then removed. Intravenous contrast was used in all patients to permit better delineation of pelvic lymph nodes and bladder. Patients were positioned with knee and feet support (Sinmed, The Netherlands) but no custom immobilization device was used. The isocenter was set in the middle of the prostate using our virtual simulation console (AdvantageSim, General Electric, Cleveland, OH) by the treating physician immediately after the first scan, while the patient waited in the treatment position on the scan table. The isocenter was then tattooed on the patient skin using our scan room mobile lasers (LAP Dorado CT-4).

# Contouring and volume definition

Structures were manually contoured on the CT scan. The clinical target volume (CTV) for the lymph nodes included the obturator, internal iliac, external (excluding the artery) iliac and sacral vessels plus a circumferential 7 mm margin in accordance with the RTOG consensus guidelines for contouring [14]. The planning target volumes (PTV) 1 was defined as the prostate and seminal vesicles (proximal part in the absence of invasion) plus a 1 cm margin in all directions except posteriorly (5 mm), plus the CTV for lymph nodes and a 7 mm set-up margin. The PTV2 consisted of the prostate gland only or the prostate gland plus invaded seminal vesicles, with a 1 cm (5 mm posteriorly) margin. The bladder was contoured in its entirety. The rectum was contoured as a whole organ but starting 2 cm above and below the CTV. Femoral heads were drawn from the top of the acetabulum to the small trochanter inferiorly. Small bowel was determined in all slices where the PTV was apparent. To take interfractional bowel motion into account, the small bowel was delineated as a whole pelvic and abdominal cavity excluding bones, muscle and other OAR, rather than contoured as individual bowel loops. For bowel, rectum and bladder, a second volume was created and defined as the considered organ minus the PTV (bowel - PTV, rectum - PTV, bladder - PTV) to avoid hot spots and improve optimization.

*4 Treatment planning by IMRT*

Treatment plans were generated using commercial software (Eclipse, Helios, version 8.2.23, Varian, Palo Alto, CA). Beam geometry consisted of 7 coplanar split fields with gantry angles of 0°, 45°, 110 °, 165 °, 195°, 250° and 325 °. IMRT was delivered using an 18-MV linear accelerator (21 EX, Varian, Palo Alto, CA) and the “sliding-window” mode of the multi-leaf collimator (MLC Millennium 120, Varian, Palo Alto, CA). Optimization process was undertaken by decreasing as much as possible the dose to OAR without altering PTV coverage, and the results were improved by modifying constraints and priority factors.

Calculation was performed with AAA algorithm, and grid of 2.5 mm.

The IMRT dose plan was normalized as needed to improve PTV2 coverage by the 74 Gy isodose, 95% of the PTV receiving at least 95% of the prescribed dose.

*5 Treatment planning by RA*

RapidArc® optimization was performed using the Eclipse software version 8.9.08 (Helios, Varian, Palo Alto, CA). A maximum dose rate of 600 MU/min and 18 MV photon beams were selected. The optimization process started with the constraints obtained with the IMRT plans. RapidArc with 1 arc (RA1) corresponded to a single 360° rotation, and RapidArc with 2 arcs (RA2) to two coplanar arcs of 360° sharing the same isocenter and optimized independently and simultaneously. These two arcs were delivered with opposite rotation (clock and counter-clock) so that off-treatment between the two beams was minimized to about 25 seconds. For RA1, field size and collimator rotation were determined by the automatic tool from Eclipse to encompass the PTV. We controlled that the collimator was rotated to a value different from zero in order to avoid the tongue-and-groove effect. For RA2, the first arc was similar to that defined in the RA1 process except for the rotation of the collimator, which was 360-X for the second arc (X corresponded to the rotation of the collimator of the first arc). To improve results, we attempted to modify constraints and priority factors of RA plans. These parameters were modified with regard to the DVH results for each patient.

For each individual patient, the same normalization was used for the RA and IMRT plans.

*6 Dose prescription and evaluation*

The prescribed doses to the PTV1 and PTV2 were delivered with a single plan using the integrated boost method to 54 Gy and 74 Gy in 37 daily fractions, respectively. This strategy allowed not only better planning results, but also potential radiobiological advantage for OAR by decreasing the dose per fraction outside the PTV2.

*7 Statistical analyses*

Doses to the PTV1, PTV2, and OAR were recorded for sliding-window IMRT and RapidArc® plans. A non-parametric Wilcoxon matched pair test was used for comparison between values of IMRT and RapidArc® for organs at risks, PTV1 and PTV2. A two-tailed p-value less than 0.05 was used to indicate statistical significance.

**Results:**

1. Contoured volumes

The mean contoured volumes (in cc ± standard deviation (SD), minimum and maximum values in brackets) were: PTV1: 906 ± 355 (419-1356); PTV2: 151 ± 56 (94-290); small bowel: 342 ± 145 (163-581); bladder: 129 ± 64 (37-239); rectum: 58 ± 19 (31-71).

1. PTV coverage

The doses received by 95% of the PTV1 (PTV1 D95) were 53.3 ± 0.9 Gy, 53.7 ± 0.9 Gy and 53.7 ± 1.3 Gy respectively for IMRT, RA1 and RA2. This means that all three techniques allowed a good PTV1 coverage by the 95% isodoses of the prescribed dose (51.3 Gy). Mean doses to PTV1 were 60.3 ± 2.1 Gy, 61.6 ± 2 Gy and 61.8 ± 2 Gy respectively for IMRT, RA1 and RA2.

Mean doses to PTV2 were 73.1 ± 0.8 Gy, 74.1 ± 0.9 Gy and 74.5 ± 0.6 Gy respectively for IMRT, RA1 and RA2. Maximal doses received by the PTV2 were 75.5 ± 1.2 Gy, 77.8 ± 1.1 Gy and 77.4 ± 0.8 Gy respectively for IMRT, RA1 and RA2, which is less than 106% of the prescribed dose (78.4 Gy).

Figure 1 shows dose volume histograms obtained with the three techniques for PTV1 and PTV2.

Figure 2 represents typical dosimetric results for static IMRT and RapidArc® plans.

1. Organs at risk sparing

Figure 1 shows dose volume histograms obtained with the three techniques for bladder, small bowel, rectum and femoral heads.

All three techniques permitted to maintain doses to OAR within dose levels recommended by the QUANTEC with a low risk of side effects [15].

For bladder, although no difference was seen for high radiation doses, there was a substantial gain in the middle doses, with mean bladder V45 values (defined as the mean percentage volume receiving 45 Gy or more, SD in brackets) of 60.0 (± 12) % for IMRT, 57.5 (± 11) % for RA1 and 52.4 (± 8) % for RA2.

Femoral heads were more irradiated with both one- and two-arc RapidArc® plans than with the IMRT plan: mean dose values (SD in brackets) (i) for the right femoral head were 14.4 (± 2.4) Gy for IMRT, 20.9 (± 4.0) Gy for RA1, and 22.8 (± 5.4) Gy for RA2 (ii) for the left femoral head 14.5 (± 1.6) Gy for IMRT, 20.3 (± 2.9) Gy for RA1, and 23.5 (± 5.2) Gy for RA2.

Both VMAT plans produced higher irradiated rectal volume at 74 Gy (hot points), but for all the dose levels below 70 Gy, this difference was in favor of the VMAT with on average a 4 Gy benefit for the median dose with RA2 as compared to static IMRT.

A significant advantage for RapidArc® was seen for the small bowel with a 2.3 Gy and a 6.4 Gy benefit on mean dose respectively for RA1 and RA2 as compared to IMRT. On average, the volume of small bowel receiving 30 Gy or more (V30) was 48 cc and 100 cc smaller respectively with RA1 and RA2 than with IMRT.

Table 1 summarizes the main and most significant results obtained with the three techniques.

1. Efficiency

RapidArc® techniques induced almost a 3-fold decrease in the number of Monitor units (MU) delivered. Of note, the number of MU is not increased when adding a second arc. Values and statistical analysis for MU are shown in table 1.

**Discussion:**

Dose levels and treatment volumes remain controversial topics for high-risk prostate cancer radiotherapy [16, 17]. However, whole pelvic irradiation is often considered in this setting, raising concerns about an increase in radiation-related toxicity. New technical developments have allowed radiation oncologists to achieve a better protection of critical organs while providing higher dose conformity to target volumes. More specifically, several studies indicated that VMAT treatment offers equal or better dosimetric results compared to static gantry IMRT when treating target volumes including the prostate gland alone or the prostate gland plus seminal vesicles [9-12]. On the contrary, only two studies have directly compared VMAT and IMRT treatment plans with pelvic lymph nodes irradiation in high-risk prostate cancer patients.

Davidson *et al.* have recently published a study assessing the role of VMAT relative to IMRT and helical tomotherapy (HT) in the management of different clinical scenarios: localized, locally advanced or post-operative prostate cancer [18]. They found that VMAT was able to improve efficiency of delivery while maintaining equivalent dosimetric quality as compared to IMRT and HT. However, this study by its nature, dealt with various clinical conditions, and yields potentially confusing results. Indeed, for each condition, the subgroups were composed of only five patients, making definitive conclusions difficult [19].

Yoo *et al.* reported the results of a dosimetric study comparing the treatment plans of ten patients with PTV including prostate, seminal vesicles and lymph nodes [13]. With a two-phase planning process (primary plan to the pelvis and additional boost plan to the prostate), they showed that IMRT reached better dose sparing for bladder, rectum and small bowel than did RapidArc®.

By contrast, our results indicate a clinically and statistically significant reduction in doses delivered to the bladder, rectum and small bowel when using RapidArc® in simultaneous integrated boost plans. A common explanation for different dosimetric results in the same setting is the variation in the definition of volumes. Nevertheless, we do not believe this could justify the major difference between these results, because our patients had overall larger PTV and smaller OAR volumes than had the patients treated in the aforementioned study*,* which led to even more complicated dosimetric plans. For example, the small bowel was delineated only on the slices where the PTV was present in our patients, whereas in the Yoo *et al.* study it was contoured up to 3 cm above the most superior slice of the PTV, where obviously there is almost no radiation dose. Another explanation could be that we focused on improving OAR sparing, even if it led to a slight increase in PTV heterogeneity, provided this heterogeneity remained between acceptable levels for each patient as defined by the ICRU 83 recommendations [20].

The last explanation could be that we used a simultaneous integrated boost method to deliver the dose to the prostate and pelvis, while Yoo *et al.* used a primary plan and a separate boost plan for each patient in their study. A single-phase process has been shown to give better results than a two-phase plan to simultaneously deliver high dose to the prostate and lower dose to the pelvic nodes in high-risk prostate cancer when using IMRT [21]. This has not been done for VMAT and the only way to answer the question will be to undertake another dosimetric study comparing simultaneous integrated boost with two-phase plans with this technique. One could criticize this single-phase approach, pointing out that the delivery of different dose levels with the same number of fractions necessarily leads to a change in the fractionation regarding the different target volumes. The way to overcome this limitation is either to hypofractionate the prostate volume while keeping a 1.8 or 2 Gy/fraction regimen on the pelvic volume, or to maintain a standard fractionation for the prostate volume while reducing the dose per fraction to the pelvis. We chose the second solution in order to achieve a lower rate of toxicity. We must assume a high degree of uncertainty regarding the α/β ratio for prostate cancer and the sensitivity of prostate tumors to fractionation, making biologically equivalent dose calculations hazardous [22-24]. However, in our study the change in fractionation probably did not impact on tumor control since it only dealt with microscopic disease (pelvic lymph nodes areas) in the absence of macroscopic involvement of lymph nodes, which would have required higher doses for cure.

Not surprisingly, two-arc plans provided better results both in terms of organ sparing and PTV coverage than single arc plans. However, both plans were acceptable, and in general superior to the static IMRT ones. Clearly, the overall treatment delivery time is longer when adding a second arc, emphasizing the need for clinicians to take this factor into consideration when choosing the most appropriate treatment plan for each individual patient.

**Conclusions:**

This study, performed in a series of ten patients, indicates that VMAT offers improved OAR sparing as compared to sliding-window IMRT while maintaining PTV coverage within acceptable levels for whole pelvis irradiation of locally advanced prostate cancer. RapidArc® improves treatment efficiency thanks to a dramatic fall in the number of MU used for irradiation.

**Acknowledgements:**

We thank Vanessa Guillaumon (Research department, CRLC Val d’Aurelle, Montpellier) who provided medical writing services.

**References:**

1. Heidenreich A, Bellmunt J, Bolla M, et al. EAU guidelines on prostate cancer. Part 1: screening, diagnosis, and treatment of clinically localised disease. Eur Urol 2011, 59:61-71.

2. Bolla M, Van Tienhoven G, Warde P, et al. External irradiation with or without long-term androgen suppression for prostate cancer with high metastatic risk: 10-year results of an EORTC randomised study. Lancet Oncol 2010, 11:1066-1073.

3. Horwitz EM, Bae K, Hanks GE, et al. Ten-year follow-up of radiation therapy oncology group protocol 92-02: a phase III trial of the duration of elective androgen deprivation in locally advanced prostate cancer. J Clin Oncol 2008, 26:2497-2504.

4. Pahlajani N, Ruth KJ, Buyyounouski MK, et al. Radiotherapy Doses of 80 Gy and Higher are Associated with Lower Mortality in Men with Gleason Score 8 to 10 Prostate Cancer. Int J Radiat Oncol Biol Phys 2011. Available at: http://www.ncbi.nlm.nih.gov/pubmed/21763081.

5. Zelefsky MJ, Pei X, Chou JF, et al. Dose escalation for prostate cancer radiotherapy: predictors of long-term biochemical tumor control and distant metastases-free survival outcomes. Eur Urol 2011, 60:1133-1139.

6. Valicenti RK, Bae K, Michalski J, et al. Does hormone therapy reduce disease recurrence in prostate cancer patients receiving dose-escalated radiation therapy? An analysis of Radiation Therapy Oncology Group 94-06. Int J Radiat Oncol Biol Phys 2011, 79:1323-1329.

7. Neuzillet Y, Négrier S, Fizazi K, et al. [The French clinical trials ongoing (GETUG and AFU) on urothelial carcinomas, kidney and prostate cancers]. Prog Urol 2010, 20 (Suppl 1):S84-89.

8. Wang JZ, Li XA, D’Souza WD, Stewart RD. Impact of prolonged fraction delivery times on tumor control: a note of caution for intensity-modulated radiation therapy (IMRT). Int J Radiat Oncol Biol Phys 2003, 57:543-552.

9. Jouyaux F, De Crevoisier R, Manens J-P, et al. [High dose for prostate irradiation with image guided radiotherapy: Contribution of intensity modulation arctherapy.]. Cancer Radiother 2010, 14:679-89.

10. Zhang P, Happersett L, Hunt M, et al. Volumetric modulated arc therapy: planning and evaluation for prostate cancer cases. Int J Radiat Oncol Biol Phys 2010, 76:1456-1462.

11. Wolff D, Stieler F, Welzel G, et al. Volumetric modulated arc therapy (VMAT) vs. serial tomotherapy, step-and-shoot IMRT and 3D-conformal RT for treatment of prostate cancer. Radiother Oncol 2009, 93:226-233.

12. Kjær-Kristoffersen F, Ohlhues L, Medin J, Korreman S. RapidArc volumetric modulated therapy planning for prostate cancer patients. Acta Oncologica 2009, 48:227-232.

13. Yoo S, Wu QJ, Lee WR, Yin F-F. Radiotherapy Treatment Plans With RapidArc for Prostate Cancer Involving Seminal Vesicles and Lymph Nodes. Int J Radiat Oncol Biol Phys 2010, 76:935-942.

14. Lawton CAF, Michalski J, El-Naqa I, et al. RTOG GU Radiation Oncology Specialists Reach Consensus on Pelvic Lymph Node Volumes for High-Risk Prostate Cancer. Int J Radiat Oncol Biol Phys 2009, 74:383-387.

15. Marks LB, Yorke ED, Jackson A, et al. Use of normal tissue complication probability models in the clinic. Int J Radiat Oncol Biol Phys 2010, 76:S10-19.

16. Al-Mamgani A, Lebesque JV, Heemsbergen WD, et al. Controversies in the treatment of high-risk prostate cancer--what is the optimal combination of hormonal therapy and radiotherapy: a review of literature. Prostate 2010, 70:701-709.

17. Morikawa LK, Roach M 3rd. Pelvic nodal radiotherapy in patients with unfavorable intermediate and high-risk prostate cancer: evidence, rationale, and future directions. Int J Radiat Oncol Biol Phys 2011, 80:6-16.

18. Davidson MTM, Blake SJ, Batchelar DL, Cheung P, Mah K. Assessing the role of volumetric modulated arc therapy (VMAT) relative to IMRT and helical tomotherapy in the management of localized, locally advanced, and post-operative prostate cancer. Int J Radiat Oncol Biol Phys 2011, 80:1550-1558.

19. Lock M, Best L, Wong E, et al. A Phase II Trial of Arc-Based Hypofractionated Intensity-Modulated Radiotherapy in Localized Prostate Cancer. Int J Radiat Oncol Biol Phys 2010. Available at: http://www.ncbi.nlm.nih.gov/pubmed/20708855.

20. The International Commission on Radiation Units and Measurements. Prescribing, recording, and reporting photon-beam intensity-modulated radiation therapy (IMRT). ICRU Report 83 J ICRU 2010, 10:1-106.

21. Li X, Wang J, Jursinic P, Lawton C, Wang D. Dosimetric advantages of IMRT simultaneous integrated boost for high-risk prostate cancer. Int J Radiat Oncol Biol Phys 2005, 61:1251-1257.

22. Bentzen SM, Ritter MA. The alpha/beta ratio for prostate cancer: what is it, really? Radiother Oncol 2005, 76(1):1-3.

23. Daşu A. Is the alpha/beta value for prostate tumours low enough to be safely used in clinical trials? Clin Oncol (R Coll Radiol). 2007, 19:289-301.

24. Nickers P, Hermesse J, Deneufbourg J-M, Vanbelle S, Lartigau E. Which α/β ratio and half-time of repair are useful for predicting outcomes in prostate cancer? Radiother Oncol 2010, 97:462-466.

**Figure legends:**

Table 1: Dosimetric results for bladder, rectum, small bowel and monitor units with the 3 techniques. P values in red means a statistically significant difference in favor of the RapidArc® treatment.

Figure 1: Mean OAR and PTV DVH plots for the ten patients with IMRT (in dashed blue), RA1 (in dashed red) and RA2 (in green). X-axis in Gray and Y-axis in percentage of the corresponding volume. RFH: right femoral head; LFH: left femoral head.

Figure 2: Typical dosimetric results for static IMRT and RapidArc® plans
